# Insights into epidemiological and clinical burden of bronchiolitis among hospitalized children: a call for preventive interventions

**DOI:** 10.3389/fped.2026.1803101

**Published:** 2026-05-28

**Authors:** Rahaf Abughosh, Fatmah Almesmari, Noora Alattar, Bilal Mohammad, Basheer Tharayil, Maimunah Uddin, Stefan Weber, Sareea AlRemeithi

**Affiliations:** 1 Pediatric Residency Program, Education Institute, Abu Dhabi, United Arab Emirates; 2 Department of Pediatrics, Sheikh Khalifa Medical City, Abu Dhabi, United Arab Emirates; 3 Purelab Reference Laboratory for Infectious Diseases, Abu Dhabi, United Arab Emirates; 4 Division of Endocrinology, Department of Pediatrics, Sheikh Khalifa Medical City, Abu Dhabi, United Arab Emirates

**Keywords:** bronchiolitis, economic burden, intensive care, length of stay, pediatrics hospitalization, respiratory syncytial virus

## Abstract

**Background:**

Bronchiolitis is a leading cause of hospitalization in infants under 2 years, with seasonal surges that strain healthcare systems. Despite its prevalence, management remains largely supportive, and predictive tools for disease severity are limited. This study aimed to characterize the clinical epidemiology, seasonal trends, and risk factors for bronchiolitis hospitalization and prolonged length of stay (LOS) at Sheikh Khalifa Medical City (SKMC), and to estimate the associated economic burden.

**Methods:**

A retrospective cross-sectional study included children aged 0–24 months admitted with bronchiolitis to SKMC between September 2023 and August 2024. Demographics, clinical features, comorbidities, laboratory findings, oxygen requirements, and escalation of care were collected from the electronic medical record. LOS, escalation to pediatric intensive care unit (PICU), and hospitalization costs were analyzed using multivariable regression models.

**Results:**

A total of 598 patients were included, representing 10.4% of pediatric admissions. The mean age was 7.8 ± 5.5 months; 59% were male, and 71% had no significant comorbidities. Respiratory syncytial virus (RSV) was identified in 47% of cases. Most were admitted for respiratory distress with normoxia (44%) or hypoxia (37%). Overall, 79.1% were managed on the ward and 14.9% in PICU; 10.8% required escalation from ward to PICU. Median LOS was 2.7 days (IQR 1.5–5), with a median cost of 21,776 United Arab Emirates Dirham (AED). Prolonged LOS was significantly associated with hypoxia, hypercapnia, comorbidities, shorter symptom duration, and secondary pneumonia. The same factors, along with RSV infection, significantly predicted PICU escalation.

**Conclusion:**

Early identification of infants with hypoxia, hypercapnia, comorbidities, or secondary pneumonia is critical to anticipate prolonged hospitalization, optimize resource planning, and guide targeted preventive strategies during seasonal peaks.

## Background

Bronchiolitis is a common lower respiratory tract infection affecting infants and young children under 2 years of age. It typically presents with cough, wheezing, crepitations, and varying degrees of respiratory distress. Respiratory syncytial virus (RSV) is the predominant causative pathogen, accounting for an estimated 60%–80% of cases worldwide (1). Despite its substantial global burden and the significant strain it places on healthcare systems during seasonal peaks, management remains primarily supportive, as no targeted antiviral therapy has yet proven effective for routine clinical use.

Globally, bronchiolitis contributes to marked seasonal surges in pediatric hospitalizations, with incidence peaking during the winter months. Studies from multiple countries, including China and Italy, consistently demonstrate this pattern, showing increases beginning in late autumn and reaching their highest levels between November and February (2). In Italy, for example, hospitalizations rise sharply in November, with peak rates reported at 7.4 cases per 1,000 children (3). These seasonal spikes create substantial strain on healthcare systems and are associated with significant economic consequences. In China, the median length of hospital stay for bronchiolitis is approximately six days, representing a considerable financial burden for families and health services (2). Similarly, Italian data indicate that hospitalization costs for bronchiolitis cases requiring pediatric intensive care are up to four times higher than costs for admissions to standard pediatric wards, underscoring the heavy economic impact of severe disease (3).

Identifying predictors of bronchiolitis severity—such as the need for intensive care or prolonged hospitalization—is essential for guiding clinical decision-making, optimizing resource allocation, and improving patient outcomes. Established risk factors include younger age at presentation, male sex, prematurity, and underlying comorbidities such as unrepaired congenital heart disease, bronchopulmonary dysplasia (BPD), neuromuscular disorders, and immunodeficiencies (1). Although numerous international studies have characterized incidence patterns, hospitalization rates, and predictors of severe disease, there is a notable scarcity of region-specific data from the Gulf region, particularly the United Arab Emirates (UAE).

This study addresses this gap by describing the clinical and epidemiological features of bronchiolitis-related hospitalizations at a major tertiary center in Abu Dhabi, examining local seasonal trends, and identifying demographic and clinical predictors of prolonged length of stay (LOS) and pediatric intensive care unit (PICU) admission. Additionally, it assesses the economic impact of bronchiolitis within this setting, providing essential local evidence that may inform targeted preventive strategies, enhance preparedness during seasonal peaks, and support future health policy planning.

## Methods

This retrospective cross-sectional study was conducted at Sheikh Khalifa Medical City (SKMC), a tertiary care hospital in Abu Dhabi, UAE. The study included children aged 0–24 months admitted with a diagnosis of bronchiolitis between September 1, 2023, and August 31, 2024. Data was obtained from the hospital's electronic medical record system (Cerner).

### Study design, setting, and population

Children with International Classification of Diseases, 10th Revision (ICD-10) diagnosis codes for bronchiolitis were eligible for inclusion, including the following ICD-10 codes: J21.0, J21.1, J21.8, J21.9. Patients with incomplete or missing key data were excluded. Extracted variables included demographic characteristics (age, sex, gestational age, birth weight), clinical presentation (duration of symptoms prior to presentation, oxygen saturation, oxygen requirement), and documented pre-existing comorbidities such as bronchopulmonary dysplasia (BPD), reactive airway disease, unrepaired congenital heart disease, neuromuscular disorders, and immunodeficiencies. Additional data included reason for admission, level of care (ward, high-dependency unit, or pediatric intensive care unit), laboratory findings (pH, pCO_2_, sodium, urea, white blood cell count, C-reactive protein), respiratory pathogen results, escalation to intensive care, and length of stay (LOS).

The telemetry unit in our hospital represents an intermediate (high-dependency) care setting, where patients receive continuous monitoring but do not require full intensive care support. Oxygen therapy in telemetry is limited to low-flow nasal cannula. Due to the retrospective design of the study, standardized clinical severity scores such as the Wang score were not applied, as they rely on subjective assessments (e.g., wheezing and respiratory distress) that were not consistently documented in the electronic medical records. Instead, objective parameters such as oxygen requirement, hypoxia, hypercapnia, and escalation of care were used as indicators of disease severity. Additionally, while detailed data on specific respiratory support modalities [e.g., high-flow nasal cannula (HFNC), continuous positive airway pressure (CPAP), non-invasive ventilation (NIV), invasive ventilation] could provide additional granularity, their use may vary based on clinician preference and institutional protocols. Therefore, in this study, escalation to PICU and need for oxygen support were used as standardized and clinically meaningful indicators of disease severity.

### Statistical analysis

All analyses were performed using Stata 18 (Version 18.5, College Station, Texas, USA). Continuous variables were summarized as means ± standard deviations, while categorical variables were expressed as frequencies and percentages. Group comparisons were performed using independent *t*-tests for continuous variables and chi-square tests for categorical variables. Multivariate logistic regression models were constructed to identify independent predictors of PICU admission and prolonged LOS. A *p*-value <0.05 was considered statistically significant.

## Results

### Demographic and clinical characteristics

**Figure 1:**
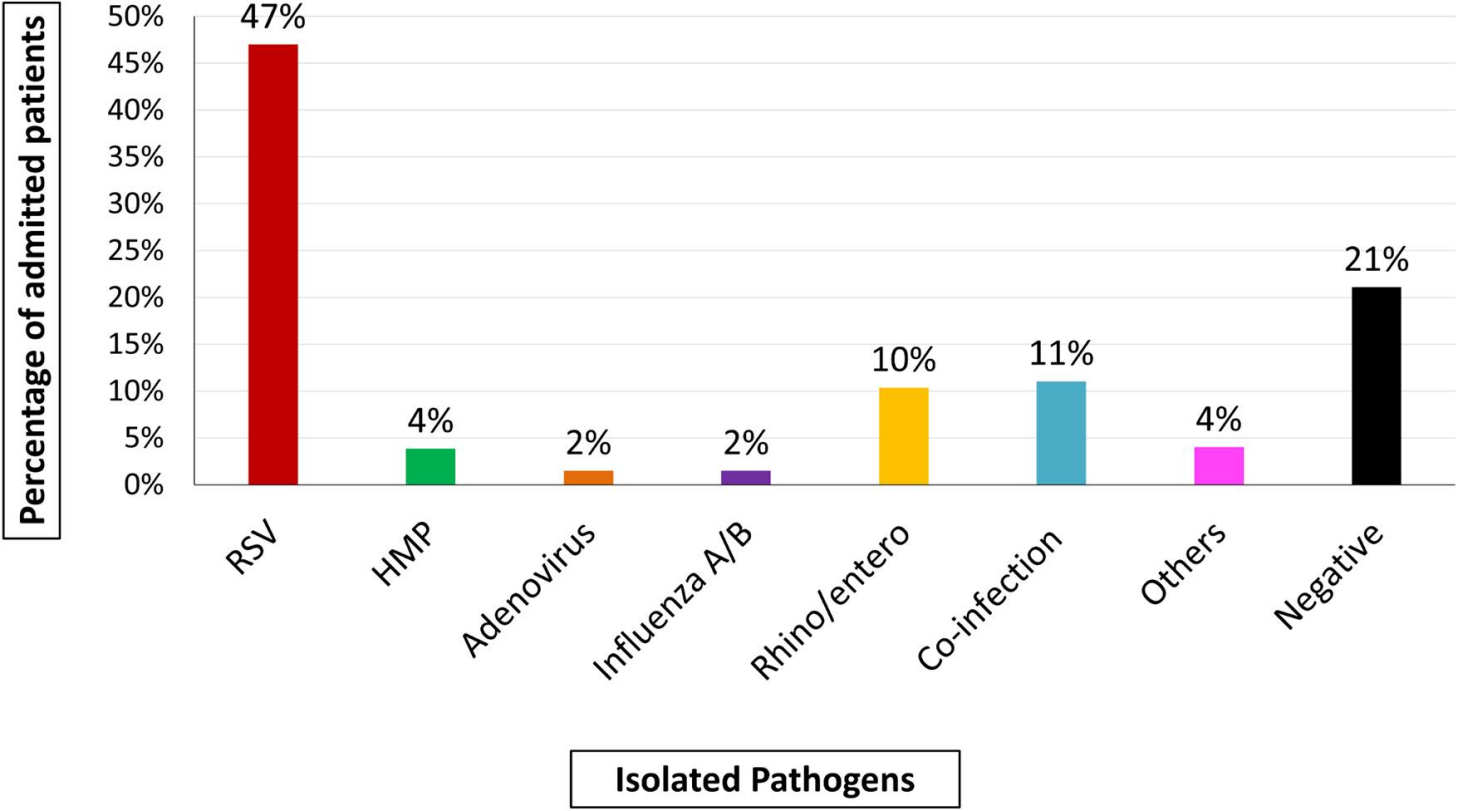
Distribution of respiratory pathogens identified among children hospitalized with bronchiolitis. HMP: Human metapneumovirus. Rhino/entero: rhinovirus/enterovirus. Co-infection: two or more viruses isolated. Others: viruses isolated not mentioned in the chart. Negative: negative respiratory viral panel.

A total of 598 patients met the inclusion criteria for analysis. The mean age at presentation was 7.8 ± 5.5 months, and 59% of the cohort were male (Table 1). Most infants (75%) were born at term, and just over half (56%) were between 3 and 12 months of age. The majority of patients (71%) had no significant underlying comorbidities. Respiratory syncytial virus (RSV) was the predominant pathogen identified and was detected in 47% of cases, excluding co-infections (Figure 1).

**Table 1:** Demographic and clinical characteristics of children aged 0–24 months hospitalized with bronchiolitis.

Total number (*n*)	598
Age at presentation in months, mean (±SD)	7.8 (±5.5)
Age group, % (*n*)	
0–3 months	24% (142)
3.1–12 months	56% (332)
12.1–24 months	20% (124)
Gender, male % (*n*)	59% (355)
Gestational age in weeks, % (*n*)	
≥37	75% (446)
34–36 + 6	14% (85)
32–33 + 6	4% (21)
28–31 + 6	4% (25)
≤28	4% (21)
Birth weight in kg, mean (±SD)	2.7 (±0.7)
Co-morbidities, % (*n*)	29% (175)
Congenital heart disease	34% (60)
Bronchopulmonary dysplasia	22% (38)
Recurrent wheeze	33% (57)
Neuromuscular disorders	10% (17)
Immunodeficiencies	1% (2)
Day of Illness, mean (±SD)	3.4 (±1.7)

**Figure 2:**
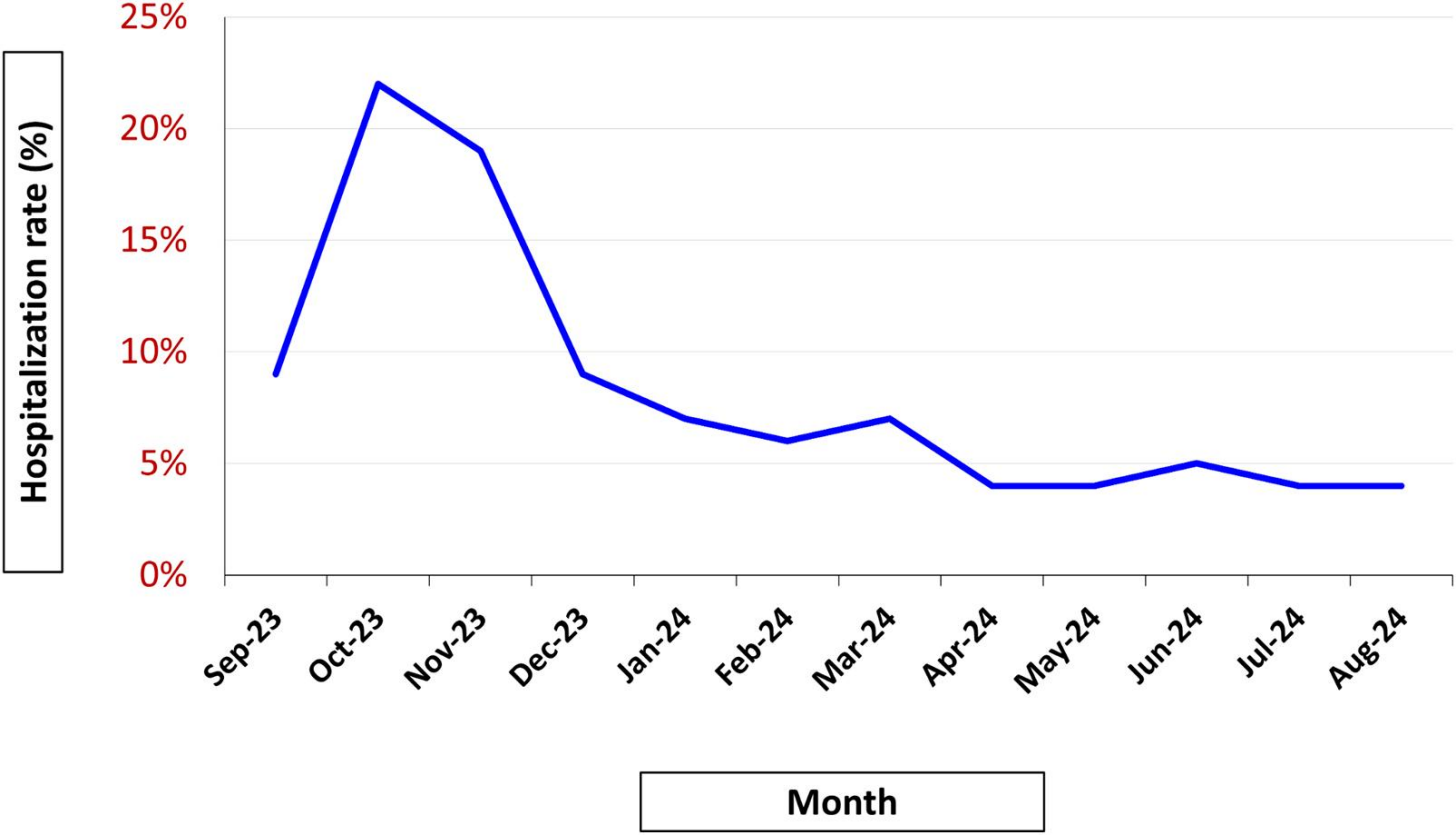
Monthly hospitalization rate for bronchiolitis from September 2023 to August 2024.

**Figure 3:**
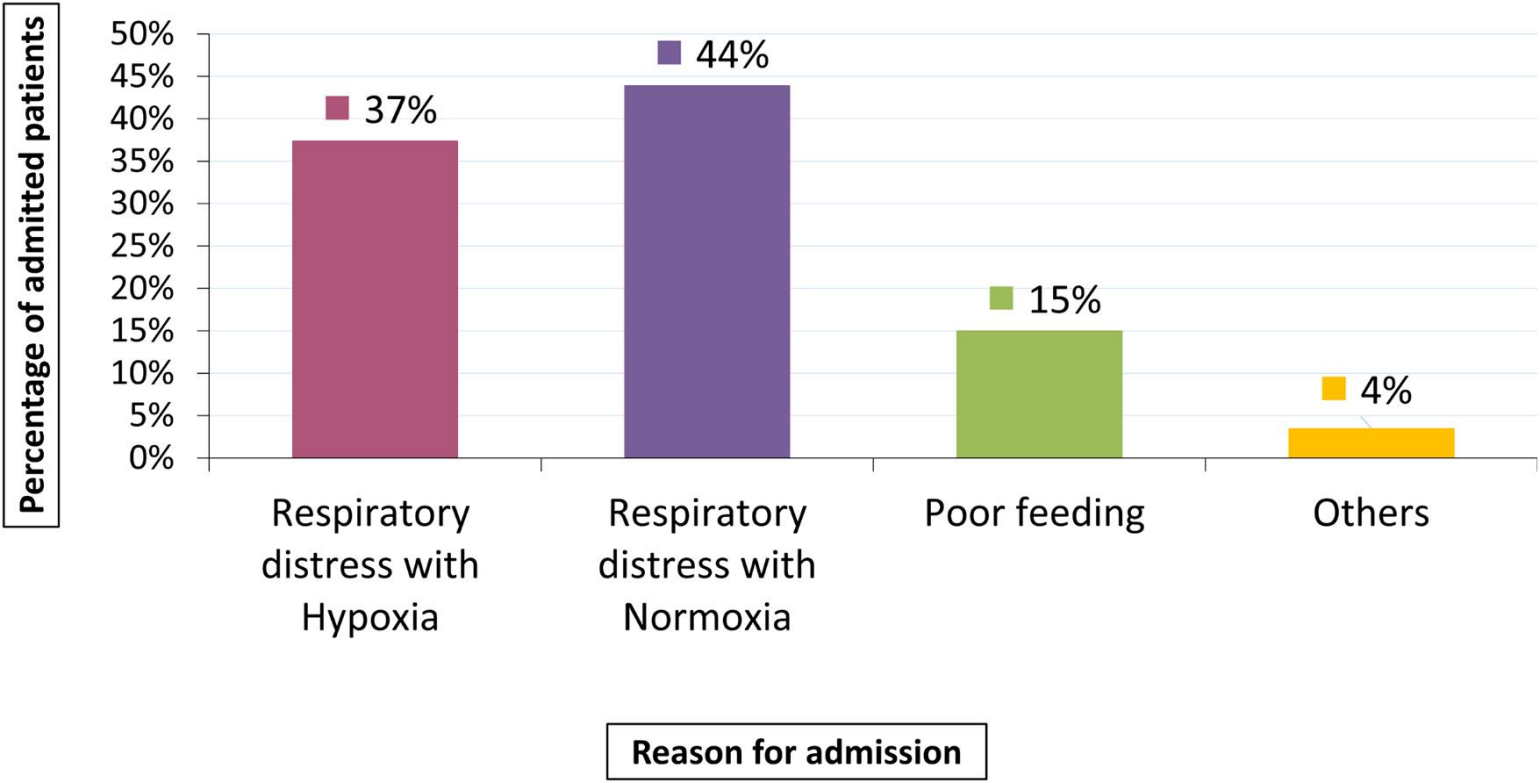
Reasons for bronchiolitis-related hospital admission among the study cohort.

**Figure 4:**
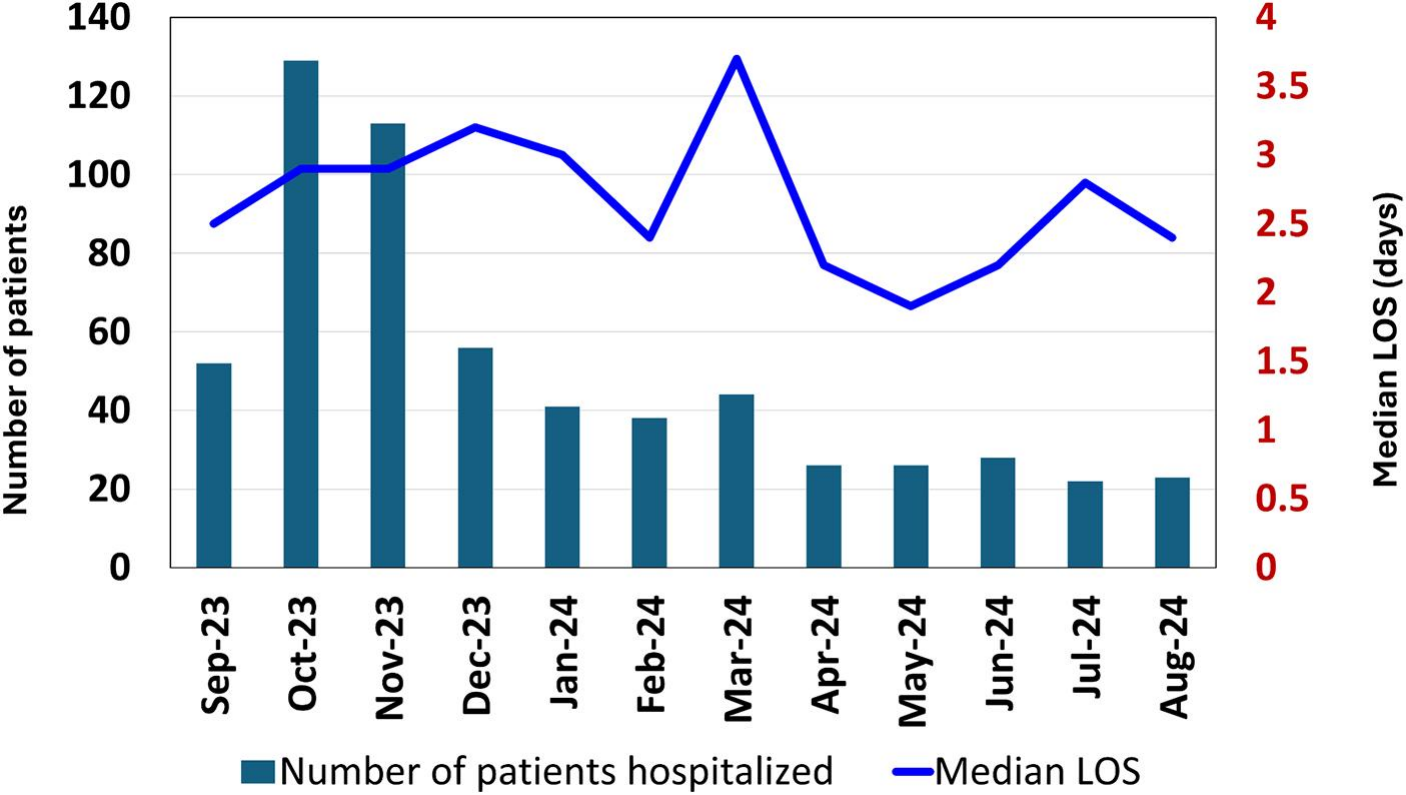
Monthly number of bronchiolitis admissions and corresponding median length of stay across the study period.

The most common reason for admission was respiratory distress with normoxia, accounting for 44% of cases, followed by hypoxia at 37% (Figure 2). Hospitalizations due to bronchiolitis demonstrated a clear seasonal trend, with a peak incidence observed in October (Figure 3). Regarding levels of care, 79.1% of patients (*n* = 473) were admitted to the regular pediatric ward, 6% (*n* = 36) to telemetry, and 14.9% (*n* = 89) to the pediatric intensive care unit (PICU). Among those initially admitted to regular or telemetry wards, 10.8% (*n* = 55) required escalation to PICU (Figure 4), contributing to a cumulative total of 144 PICU bed-days utilized throughout the study period.

### Prevalence, length of stay, and predictors of severity

**Figure 5:**
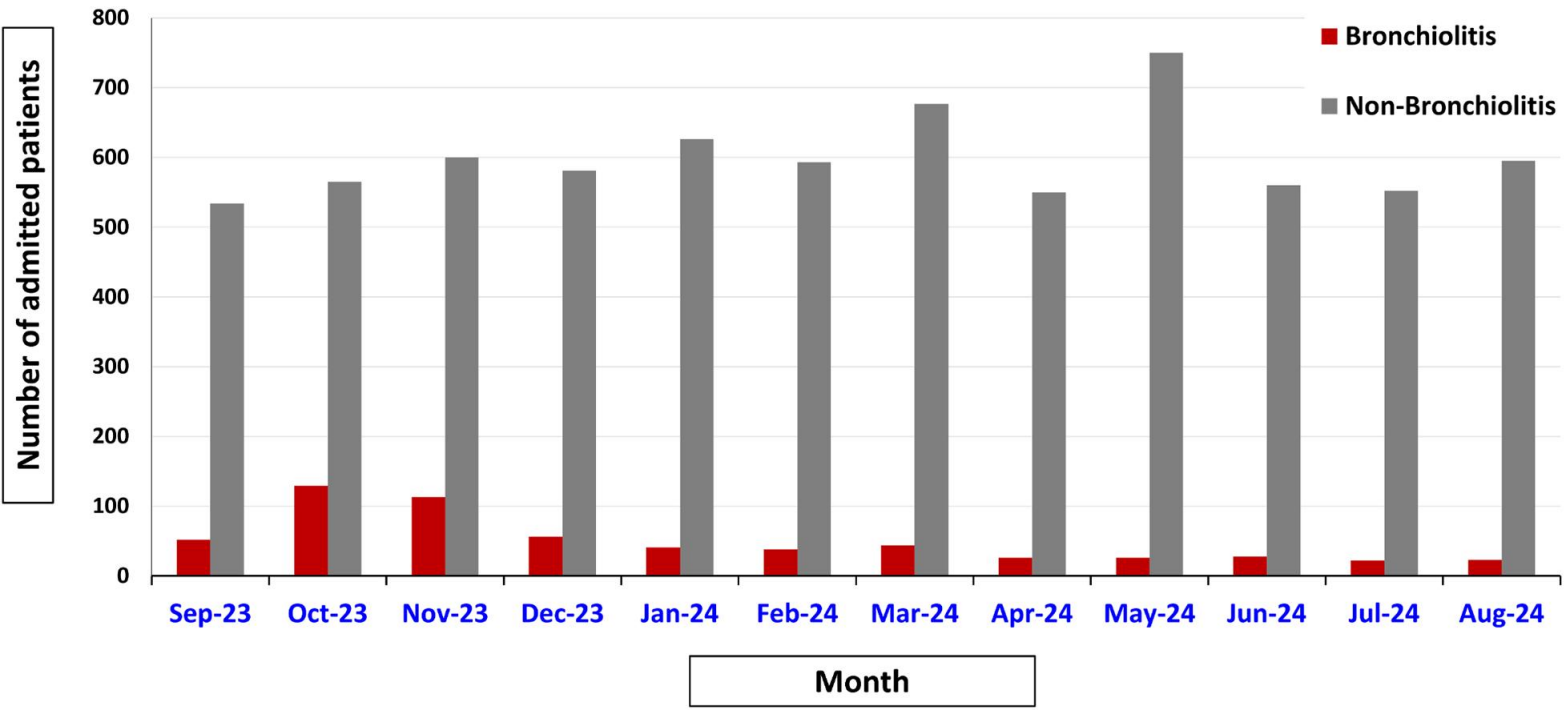
Monthly bronchiolitis admissions compared with non-bronchiolitis pediatric admissions.

**Figure 6:**
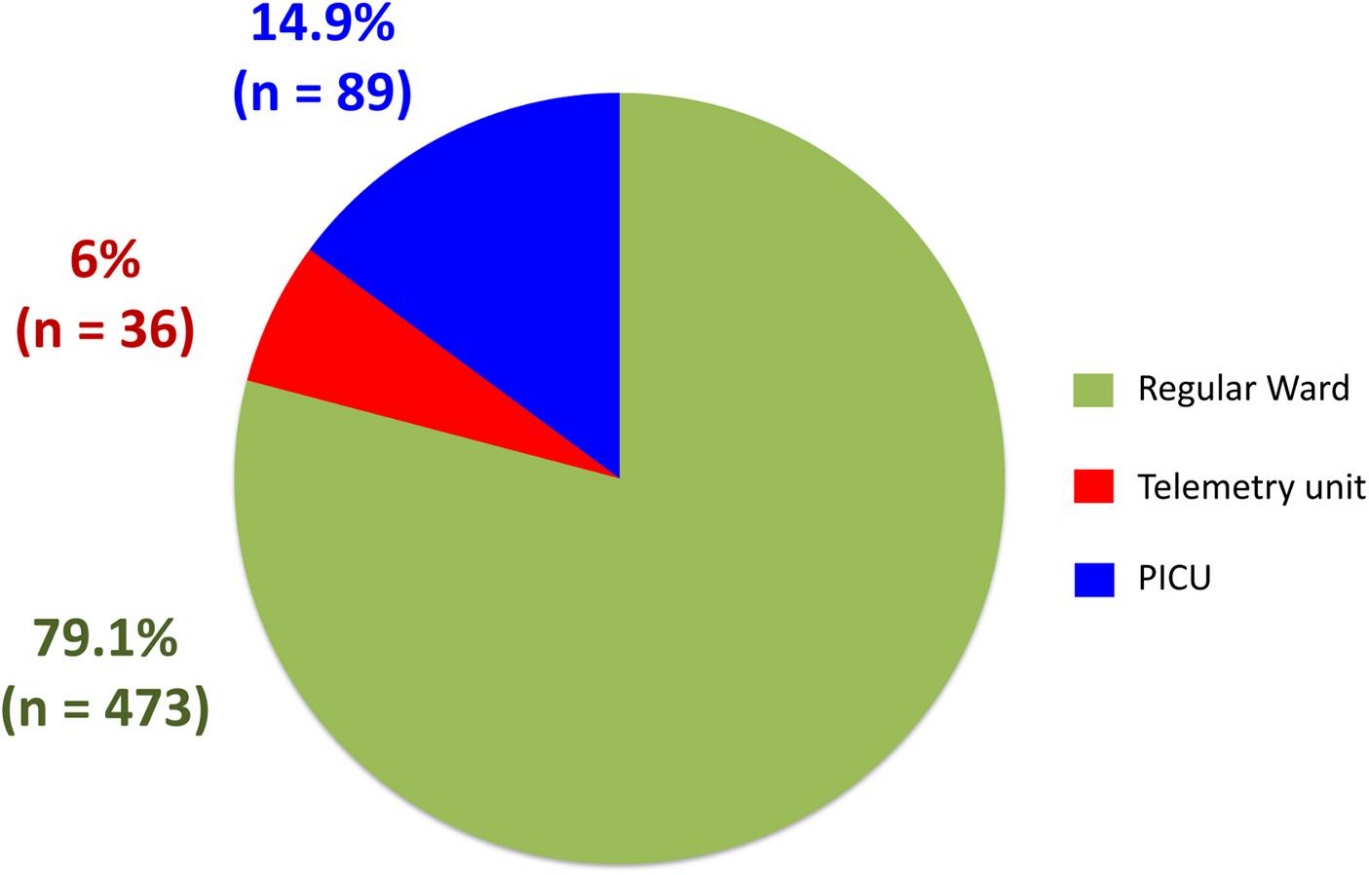
Distribution of patients according to level of care: regular ward, telemetry unit, and pediatric intensive care unit.

Bronchiolitis represented 10.4% of all pediatric admissions during the study timeframe. Monthly bronchiolitis admissions relative to overall pediatric admissions are displayed in Figure 5. The overall median length of stay (LOS) was 2.7 days (IQR 1.5–5). Patients requiring PICU admission experienced substantially longer hospitalizations, with a median LOS of 6.6 days (IQR 4.5–9.6). Table 2 summarizes the median LOS across different age categories and comorbidity groups, while Figure 6 illustrates fluctuations in LOS and case volume across the study months.

**Table 2:** Median length of stay stratified by PICU admission, age group, and presence of comorbidities.

LOS	Median (IQR)
All subjects	2.7 (1.5–5)
PICU admission	6.6 (4.5–9.6)
Non-PICU admission	2.2 (1.4–3.6)
Age group	
0–3 months	3.0 (1.6–5.1)
3.1–12 months	2.8 (1.6–5.4)
12.1–24 months	2.5 (1.6–4.7)
Comorbidities	
Yes	3.6 (1.2–7.8)
No	2.6 (1.5–4.5)

**Table 3:** Multivariable regression analysis for predictors of PICU admission.

	Total number	
Variables	Odds ratio (OR)	*p* value
Gestational age	0.969	0.424
Age at presentation		
0–3 months	1	
3.1–12 months	0.742	1.355
12.1–24 months	0.740	1.569
Gender	1.487	0.923
Co-morbidities	1.067	0.832
Duration of Illness before admission (days)	0.754	0.001
Re-admission (within 30 days)	1.731	0.139
Previous bronchiolitis	0.627	0.138
Oxygen saturation	0.915	0.000
pCO_2_ level	1.035	0.031
RSV positive	1.865	0.018
Secondary pneumonia	3.332	0.000

Multivariable regression analysis identified several significant predictors of disease severity. Factors independently associated with escalation to PICU included the presence of hypoxia at admission, elevated pCO_2_ levels (hypercapnia), shorter symptom duration prior to presentation, RSV positivity, and the presence or development of Secondary pneumonia, as outlined in Table 3. Similarly, predictors of prolonged LOS included hypoxia and hypercapnia at presentation, existing comorbidities, shorter symptom duration, and secondary pneumonia, as detailed in Table 4. Together, these findings highlight consistent clinical variables that correlate with both increased risk of deterioration and extended hospitalization.

**Table 4:** Multivariable regression analysis for predictors of prolonged length of stay.

	Total number	
Variables	Coefficient (*r*)	*p* value
Gestational age	0.080	0.260
Age at presentation		
0–3 months	0	
3.1–12 months	−0.268	0.635
12.1–24 months	1.159	0.093
Gender	−0.678	0.115
Co-morbidities	2.207	0.000
Duration of Illness before admission (days)	−0.289	0.028
Re-admission (within 30 days)	0.697	0.308
Previous bronchiolitis	0.009	0.986
Oxygen saturation	−0.104	0.007
pCO_2_ level	0.096	0.001
RSV positive	0.781	0.087
Secondary pneumonia	2.023	0.000

### Financial burden

The economic impact of bronchiolitis during the study period was considerable. Table 5 outlines the average direct cost of bronchiolitis-related hospitalization per patient per month, expressed in UAE Dirhams [1 USD = 3.7 United Arab Emirates Dirham (AED)]. The median cost of hospitalization was 21,776 AED (IQR 18,175–25,377) per patient. When aggregated across all admissions, the total direct healthcare expenditure associated with bronchiolitis exceeded 13 million AED. Costs were highest during the seasonal peak from September to December, underscoring the substantial financial strain imposed by bronchiolitis during high-incidence months.

**Table 5:** Monthly direct hospitalization costs for bronchiolitis admissions in AED.

Month	Year	Bronchiolitis cases	Direct sum costs	Average cost per case
September	2023	62	1,127,019	18,177.73
October	2023	154	3,413,430	22,165.13
November	2023	123	2,253,387	18,320.22
December	2023	72	2,150,620	29,869.72
January	2024	48	1,248,786	26,016.38
February	2024	56	1,134,706	20,262.61
March	2024	50	1,071,329	21,426.58
April	2024	39	1,678,943	43,049.82
May	2024	36	1,321,918	36,719.94
June	2024	35	728,579	20,816.54
July	2024	31	685,910	22,126.13
August	2024	39	457,874	11,740.36

## Discussion

This study provides the first comprehensive evaluation of the clinical epidemiology, disease severity, and healthcare burden of bronchiolitis-related hospitalizations at a major tertiary care center in Abu Dhabi. The observed seasonal trend, with hospitalization rates peaking between September and December, mirrors patterns reported in studies from Italy, China, and the United States (3). This early seasonal rise also reflects the post-coronavirus disease 2019 (COVID-19) shift in RSV circulation documented globally, underscoring the importance of region-specific surveillance.

Among the most important findings, oxygen support at presentation emerged as a strong predictor of both prolonged hospital stay and escalation to intensive care. This aligns with previous literature identifying early hypoxia as a key marker of disease severity (1). Children requiring oxygen support had a mean LOS more than twice that of those who did not, highlighting the prognostic significance of early respiratory compromise. Comorbidities—particularly congenital heart disease and chronic lung disease—were also associated with increased LOS, reaffirming the heightened vulnerability of medically complex infants. Younger age correlated with longer hospitalization, consistent with established evidence that young infants experience more severe bronchiolitis.

RSV was the predominant pathogen in our cohort, detected in 47% of cases as a single pathogen and in 55% when co-infections were included. RSV positivity was also an independent predictor of PICU admission, emphasizing the need for targeted RSV prevention strategies. Current guidelines from the American Academy of Pediatrics (AAP), Centers for Disease Control and Prevention (CDC), and Infectious Diseases Society of America (IDSA) recommend prophylaxis for high-risk infants—particularly those born prematurely, those with bronchopulmonary dysplasia, or those with significant congenital heart disease (4, 5). Palivizumab remains the standard preventive agent, targeting the RSV fusion protein to inhibit viral entry. In the UAE, Palivizumab is widely used according to international recommendations, with monthly dosing typically initiated before the onset of the local RSV season (6).

Importantly, our data indicates a marked rise in hospitalizations beginning in October, suggesting that initiating Palivizumab prophylaxis by late September may offer improved protection. Although Palivizumab has demonstrated a 55% reduction in RSV-related hospitalizations among high-risk infants (7), its monthly dosing schedule and high-cost limit broader implementation.

The majority of children in our cohort, however, were term infants with no comorbidity and therefore ineligible for Palivizumab under current guidelines. This highlights a large population left vulnerable to RSV infection. The recent approval of Nirsevimab represents a major advance, offering season-long protection with a single dose for all infants, regardless of risk status. Data from the HARMONIE trial demonstrated an 83.4% reduction in RSV-related hospitalizations (8), positioning Nirsevimab as a promising tool for population-level immunoprophylaxis.

Maternal vaccination has also emerged as an effective complementary strategy. Pfizer's RSVpreF vaccine (Abrysvo), administered during late pregnancy, provides passive immunity to newborns through transplacental antibody transfer. Clinical trials report a 57.3% reduction in medically attended RSV-related lower respiratory tract illness and a 48.2% reduction in RSV-related hospitalizations in the first 6 months of life (5). The vaccine has been approved for maternal use in several countries, including the UAE (6), and offers a practical mechanism to protect infants during their most vulnerable early months.

From an economic perspective, bronchiolitis constituted 10% of all pediatric admissions in our center and contributed to a median hospitalization cost of $5,879.52 per patient. The annual direct healthcare expenditure exceeded $3.5 million. These figures underscore the substantial financial burden imposed by this condition, particularly during peak months. Preventive strategies such as maternal vaccination, immunoprophylaxis for high-risk infants, and implementation of newer long-acting monoclonal antibodies could significantly reduce hospitalizations and associated costs (9).

Emerging agents such as Clesrovimab (MK-1654), a long-acting monoclonal antibody currently under investigation, show additional promise. Phase 2b/3 trial data demonstrates reductions of 91.7% in severe medically attended RSV infections and 90.9% in RSV-related hospitalizations (8). With a single-dose regimen and a safety profile similar to placebo, Clesrovimab may soon offer another effective preventive option pending regulatory approval (10).

Cost-effectiveness analyses provide further insight into the implementation of new preventive strategies. A recent U.S. study estimated an incremental cost-effectiveness ratio of $153,517 per quality adjusted life year (QALY) gained for Nirsevimab during the first RSV season, with higher Incremental Cost-Effectiveness Ratios (ICERs) in children at increased risk of hospitalization. The analysis highlighted that RSV hospitalization costs, drug pricing, and QALY losses substantially influenced outcomes. The study concluded that Nirsevimab may be cost-effective, particularly among higher-risk infants (6). These findings support consideration of RSV immunization strategies tailored to local epidemiology and economic context.

Collectively, the evidence underscores the urgent need for a multifaceted approach to RSV prevention, including earlier initiation of prophylaxis, maternal vaccination, and adoption of emerging long-acting monoclonal antibodies. Integrating these strategies into clinical practice, informed by region-specific data such as ours, will be essential for reducing the clinical and economic burden of bronchiolitis (4–6, 9, 11).

## Limitations

This study has several limitations. First, its retrospective design may be subject to incomplete data capture and potential documentation bias. Second, as a single-center study conducted at a tertiary care hospital, the findings may not be fully generalizable to other healthcare settings or regions within the UAE. Additionally, certain clinical variables that may influence disease severity, such as environmental factors and detailed socioeconomic data, were not available in the electronic medical records.

Finally, while we estimated the direct healthcare costs of hospitalization, indirect costs such as parental work loss and long-term healthcare utilization were not assessed. Despite these limitations, the study provides valuable insight into the clinical and economic burden of bronchiolitis in a large cohort of hospitalized children in the UAE.

## Conclusion

In this study, bronchiolitis accounted for 10.4% of pediatric admissions, with a median length of stay of 2.7 days and a median hospitalization cost of 21,776 AED per patient, highlighting a substantial clinical and economic burden. Key predictors of disease severity included hypoxia and hypercapnia on admission, presence of co-morbidities, shorter duration of symptoms prior to presentation, and development of secondary pneumonia, all of which were associated with prolonged length of stay and increased likelihood of PICU admission. RSV was the most frequently identified pathogen and was independently associated with escalation of care, reinforcing its role in severe disease.

These findings provide region-specific data on bronchiolitis burden in the UAE and may inform future healthcare planning, including resource allocation during peak seasons and the implementation of preventive strategies such as RSV immunoprophylaxis. Targeted preventive strategies such as earlier vaccination with Palivizumab prior to peak months should be considered. Additionally, the use of maternal RSV vaccine (RSVpreF), must be encouraged. Moreover, future prospective studies are needed to evaluate the long-term outcomes and cost-effectiveness of emerging RSV preventive strategies, such as Nirsevimab, in the UAE and their integration into local immunization programs.

## Data Availability

The raw data supporting the conclusions of this article will be made available by the authors, without undue reservation.
